# Supplementary material for: A Framework for the Computational Linguistic Analysis of Dehumanization
Source: Front Artif Intell. 2020 Aug 7;3:55. doi: 10.3389/frai.2020.00055 (PMC7861242; doi:10.3389/frai.2020.00055)
Supplement: Supplementary file 1 [file Data_Sheet_1.PDF]

Supplementary Material

1 SUPPLEMENTARY TABLES AND FIGURES

Label	Count	Label	Count	Label	Count	Label	Count
gay(s)	96977	lgbt	1783	lgbtq	129	agender	10
lesbian(s)	20233	transvestite(s)	625	glbt	68	aromantic	5
homosexual(s)	16638	tran(s)sexual(s)	627	genderqueer	51	lgbtqia	4
transgender(s/ed)	6066	asexual	255	lgb	29	genderfluid	0
bisexual(s)	3464	intersex	210	pansexual	22	lgbtqqia	0

Table S1. Overall counts for all LGBTQ terms in the New York Times from 1986-2015. Each label includes its morphological and orthographic variants.

1.1 Nearest neighbor valence with different thresholds

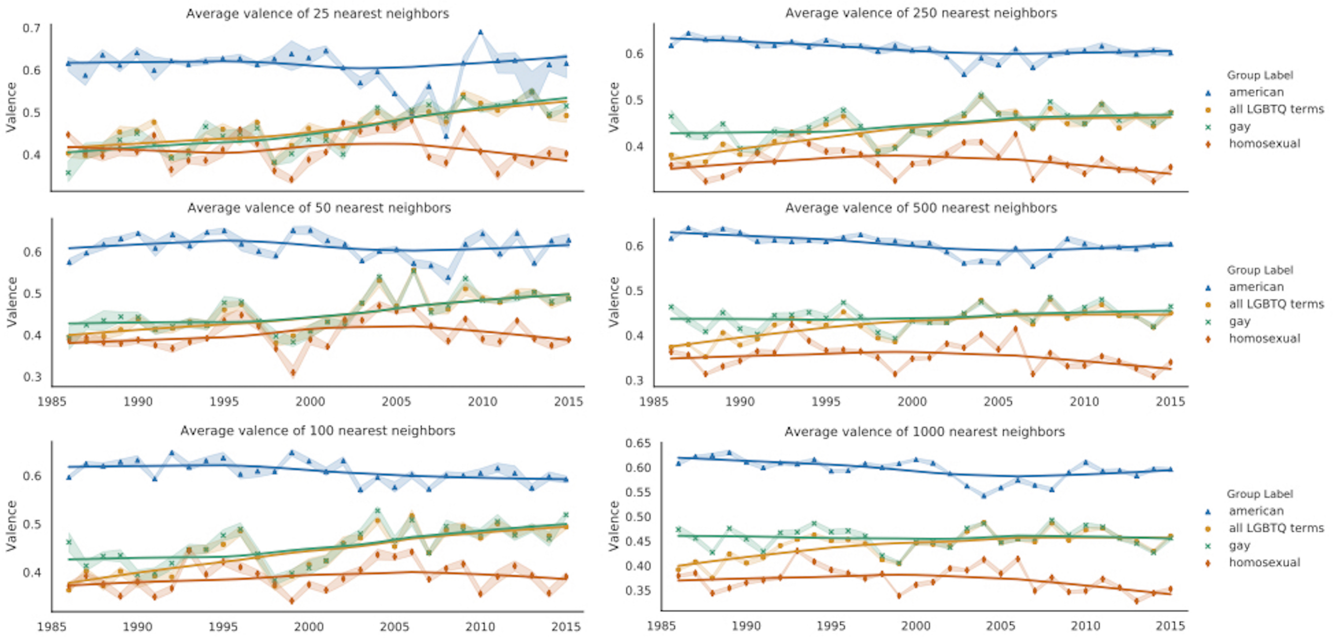

Figure S1. Average valence of 25, 50, 100, 250, 500 and 1000 nearest words to vector representations of *gay*, *homosexual*, *all LGBTQ terms*, and *American*, averaged over 10 word2vec models trained on *New York Times* data from each year. The solid lines are Lowess curves for visualization purposes. Words' valence scores are from the NRC VAD Valence Lexicon. For all plots, the shaded bands represent 95% confidence intervals.

1.2 Nearest neighbor dominance with different thresholds

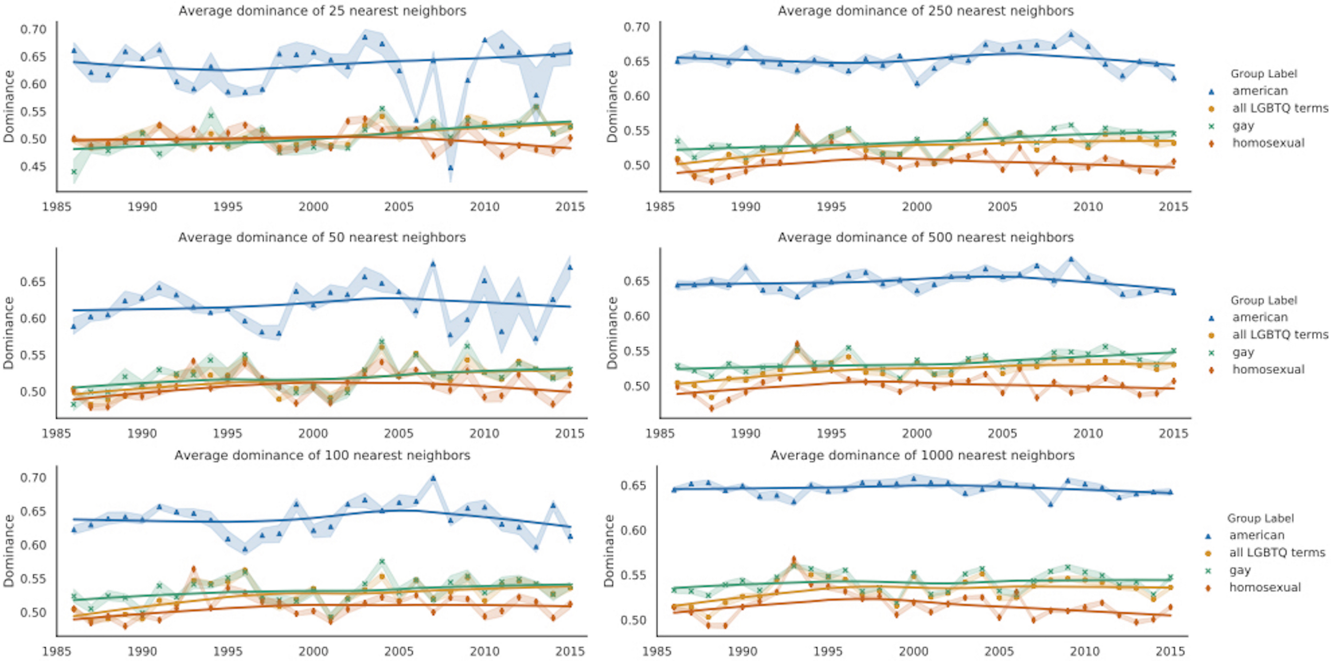

Figure S2. Average dominance of 25, 50, 100, 250, 500 and 1000 nearest words to vector representations of *gay*, *homosexual*, *all LGBTQ terms*, and *American*, averaged over 10 word2vec models trained on *New York Times* data from each year. The solid lines are Lowess curves for visualization purposes. Words' dominance scores are from the NRC VAD Dominance Lexicon. For all plots, the shaded bands represent 95% confidence intervals.

1.3 Valence prediction results

In addition to quantifying the *negative evaluation of a target group* by calculating the average valence score of a group label's vector representation's nearest neighbors according to the NRC VAD lexicon, we also directly induced a valence score from the vector representation itself. We use the zero-centered, normalized, word embeddings created for each year as features in a regression model to directly predict valence (Field et al., 2019). Specifically, we train ridge regression models for each year, where each year's Word2Vec representation for words from the NRC VAD lexicon are features and the lexicon's valence scores are labels. 85% of words from the VAD lexicon were kept as the training set, and the remaining 15% was used as a test set to evaluate performance. We then use this set of regression models to predict a group label's valence from its vector representation.

Figure S3 shows the directly-induced valence score for each set of group labels from the ridge regression fit to the NRC VAD valence lexicon. Because we trained a different Word2Vec model for each year, we trained a different ridge regression model for each year. Over all thirty years, the Pearson correlation between predicted valence and actual valence on the test set ranged from 0.617 to 0.675, and R^2 values ranged from 0.423 to 0.451.The predicted scores show similar trends to the average neighbor valence. *Homosexual* has the most negative valence for every year, followed by *gay* and the aggregate over all LGBTQ terms, followed by *American* with the most positive valence. *American* is significantly more positive than all LGBTQ labels over all years (Wilcoxon signed-rank test; $p < 0.0001$), and *gay* is significantly more positive than *homosexual* for every year (Wilcoxon signed-rank test; $p < 0.0001$). Despite the stability in differences between these terms across experiments, the regression analysis suggests different temporal dynamics. Figure S3 shows that the predicted valence for *gay*, *homosexual*, and all LGBTQ terms all increase over time, but *homosexual*'s predicted valence decreases from 2001 to 2015 ($p < 0.01$). This result is consistent with the other findings in this article in illustrating the pejoration of *homosexual* in recent years.

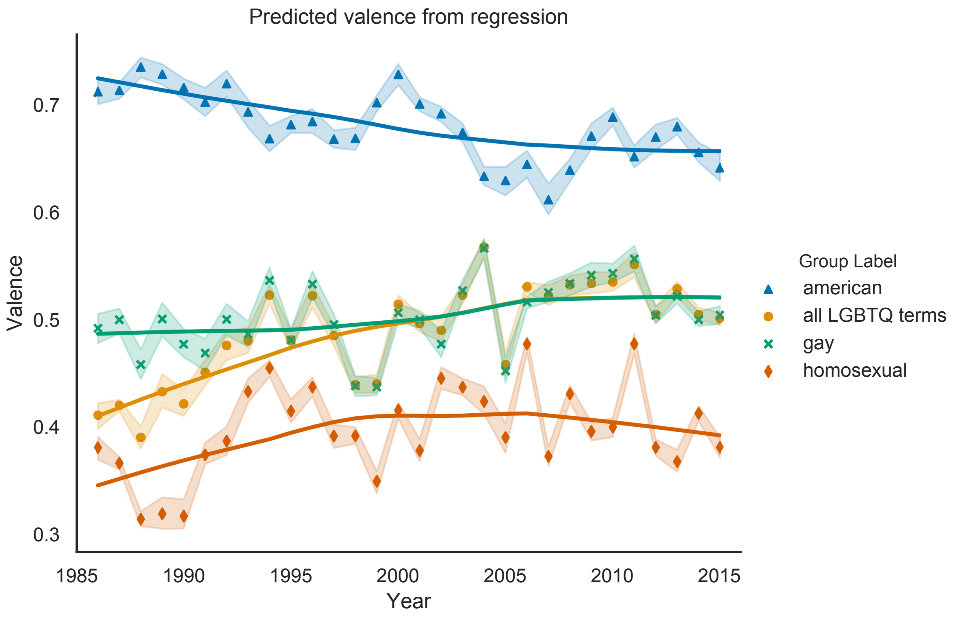

Figure S3. Predicted valence of our representations of *gay*, *homosexual*,*all LGBTQ terms*, and *American* directly induced by fitting ridge regression models to the NRC VAD Valence Lexicon with the lexicon's words' Word2Vec vectors as features for each year. Results are averaged over 10 Word2Vec models trained over each year's data. Shaded bands represent 95% confidence intervals and the solid lines are Lowess curves as visual aids. Higher scores represent more positive valence.

1.4 Agency prediction results

Because we use the NRC VAD dominance lexicon to quantify *denial of agency* in the same way we use the valence lexicon to quantify *negative evaluations of a target group*, we again directly induce scores directly from target group label representation. We train another set of ridge regression models with word embedding features for each year, but now we fit the model to the NRC VAD dominance lexicon's scores rather than the valence scores.

Figure S4 shows the predicted dominance for each group label, which is calculated by fitting ridge regression models to the NRC VAD Dominance Lexicon using the lexicon's words' Word2Vec representations as features for each year. Pearson correlations between predicted and actual dominance scores for all regression models ranged from 0.561 to 0.614 on the test set, and R^2 values range from 0.338 to 0.361. Consistent with the average neighbor dominance approach , *American* has significantly greater dominance than any of the other LGBTQ terms (Wilcoxon signed-rank test; $p < 0.0001$). However a Wilcoxon signed-rank test over each year's means shows that there is no significant difference between the terms *gay* and *homosexual* ($p = 0.21$). The predicted dominance of *all LGBTQ terms* and *gay* significantly decrease ($p < 0.0001$), but not in the last 15 years ($p = 0.85$ for *all LGBTQ terms* and $p = 0.51$ for *gay*). *Homosexual* does not significantly change in predicted dominance in either the full 30 years ($p = 0.96$) or in the last 15 years ($p = 0.89$).

Why do *gay* and *homosexual* show such different patterns in directly-induced predicted dominance from the regression than average dominance based on their neighbors' entries in the NRC VAD lexicon? While the average dominance over the nearest neighbors showed significant differences, they were small in magnitude (often corresponding to differences of less than 0.025 points on a scale from 0 to 1). Perhaps because the word2vec features could only predict just over a third of the variance in dominance scores, they were not able to capture subtle semantic distinctions that could characterize differences in dominance scores

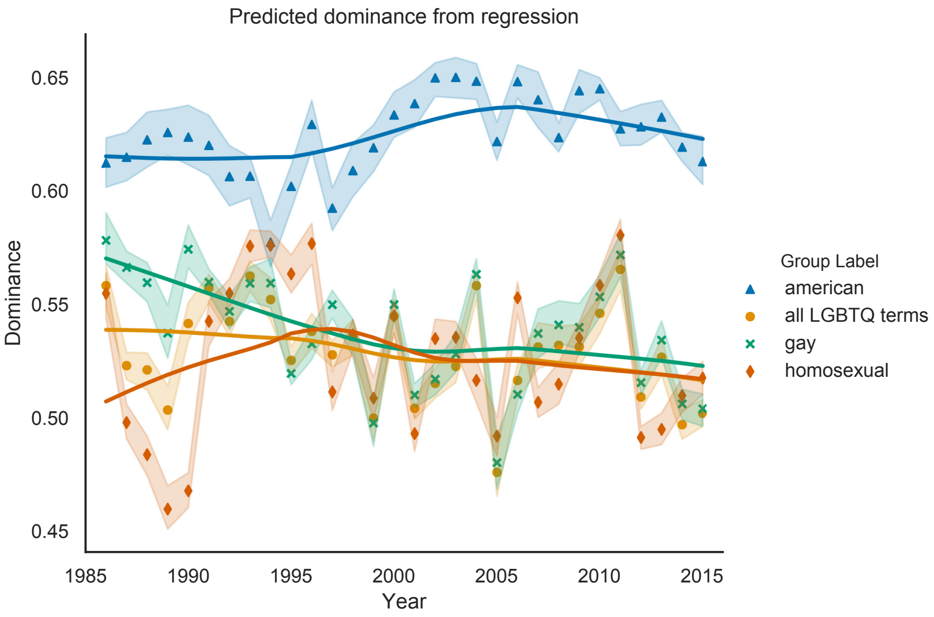

Figure S4. Predicted dominance of our representations of *gay*, *homosexual*, *all LGBTQ terms*, and *American* directly induced by fitting ridge regression models to the NRC VAD Dominance Lexicon with the lexicon's words' Word2Vec representations as features for each year. Results are averaged over 10 Word2Vec models trained over each year, shaded bands represent 95% confidence intervals and the solid lines are Lowess curves for visualization purposes. Higher scores represent greater predicted dominance.

1.5 Precision, Recall, and F1 evaluation metrics

Component	Precision	Recall	F1
negative evaluation	0.703	0.696	0.698
denial of agency	0.547	0.547	0.547
moral disgust	1.000	0.63	0.773
vermin	0.789	0.778	0.779

Table S2. Precision, recall, and weighted F1 scores for each of our four vector-based experiments. Each paragraph was labeled as "not dehumanizing" if the majority of annotators rated the paragraph above the overall median rating (4 for moral disgust and 3 for each other component) , or "dehumanizing" if the majority of annotators rated the paragraph below the overall median rating. Remaining paragraphs were discarded for these calculations.
